# In *Drosophila* Hair Development, Shavenbaby Is at the Beginning of the End

**DOI:** 10.1371/journal.pbio.0040310

**Published:** 2006-08-22

**Authors:** Richard Robinson

In the past two decades, the grand strategy of animal development has become clear: initial sets of transcription factors turn on some genes and turn off others, creating new sets in turn, at each step defining the fate of cells ever more precisely as embryonic development proceeds. At the end of this process, a fully differentiated cell with a characteristic shape emerges, but the signals that bring about these final steps have often remained elusive. A new study by Hélène Chanut-Delalande, Serge Plaza, and colleagues deciphers these signals for epidermal hair formation in *Drosophila*, illuminating the link between the cascade of transcription factors and the production of a specific cell shape in this model animal.

The embryo of the fruit fly is divided into parallel segments. The epidermal surface of each segment may be smooth, or studded with projections known as trichomes. Called denticles on the ventral surface and hairs on the dorsal surface, trichomes arise from extensions of the cytoplasm of individual cells, and are filled with the cytoskeletal protein actin. Previous work has shown the importance of several transcription factors in trichome formation, converging on Shavenbaby (Svb), the most “downstream” regulator of trichomes yet identified (*svb* mutants do not form trichomes, giving the embryos a “shaven” look).

To find the downstream targets of Shavenbaby, the authors examined gene expression patterns in *Drosophila* epidermis, looking for genes whose expression correlated in space and time with that of trichome formation. A gene called *miniature* matched the pattern closely. They showed that *svb* mutation abolished *miniature* expression, as did a repressor of *svb* activity. When they expressed *svb* in cells where it is normally silent, *miniature* was also expressed. And in a species with restricted *svb* expression, the pattern of restriction was matched by restricted *miniature* expression as well. The sine qua non of a transcription factor is its ability to directly interact with its target DNA. The authors showed that the Svb protein was indeed able to bind with a small region of the *miniature* gene, influencing its transcription.

But is *miniature* the only gene Shavenbaby controls in trichome formation? When deleted, denticles still form, but are misshapen, and when expressed where it is normally silent, it was not sufficient to form denticles by itself. From this, the authors deduced that Shavenbaby must have other targets besides *miniature* that control denticle formation. Beginning with database searches and continuing with molecular analysis, they found a small handful of genes specifically activated by Shavenbaby and involved in formation of denticles, each of which helps control dynamics of actin reorganization in the epidermis. No single gene mutation abolished denticle formation, but if all were mutated, denticles (or dorsal hairs) were either tiny and misshapen, or altogether absent, suggesting that collectively, the identified genes were in charge of trichome formation. *miniature*, their experiments showed, does not control actin dynamics, but acts at the epidermal cell membrane to regulate the interaction of cytoskeletal elements with the overlying hard cuticle layer. Thus, Shavenbaby controls both actin-related genes and at least one other gene critical for formation of the final shape of the epidermal cell. Finally, the authors showed that Shavenbaby also helps control pigmentation of denticle cells, through regulation of a gene in the pigment synthesis pathway.

**Figure:**
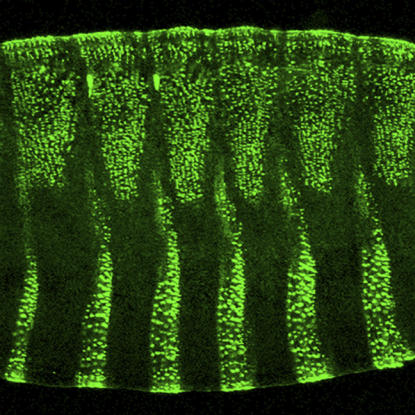
Cell shape remodeling: analyzing the function of genes specifically expressed in cellular extensions of *Drosophila* embryonic epidermis (green stain of a protein called Miniature).

From these results, the authors propose that Shavenbaby regulates a “morphological module” that directly influences epidermal form. They note that Shavenbaby's role in forming both denticles and dorsal hairs, which have different shapes, indicates the flexibility of the module, suggesting the module may be used elsewhere as well, and that the fine tuning that produces one or another type of trichome is likely done by elements both up- and downstream from Shavenbaby. It is likely that other genes, yet to be identified, are also regulated by Shavenbaby, and that some or most of these may be involved in actin remodeling or other aspects of epidermal shape determination. And now, researchers have a platform for investigating these questions.

